# Electric field simulation and appropriate electrode positioning for optimized transcranial direct current stimulation of stroke patients: an in Silico model

**DOI:** 10.1038/s41598-024-52874-y

**Published:** 2024-02-03

**Authors:** Mi-Jeong Yoon, Hye Jung Park, Yeun Jie Yoo, Hyun Mi Oh, Sun Im, Tae-Woo Kim, Seong Hoon Lim

**Affiliations:** 1 Department of Rehabilitation Medicine, College of Medicine, St. Vincent’s Hospital, The Catholic University of Korea, Seoul, Republic of Korea; 2 Department of Rehabilitation Medicine, College of Medicine, Seoul St. Mary’s Hospital, The Catholic University of Korea, 222 Banpo-daero, Seocho-Gu, Seoul, 06591 Republic of Korea; 3 Department of Rehabilitation Medicine, National Traffic Injury Rehabilitation Hospital, Jungang-Ro 260, Yangpyeong-EupGyeongki-Do, Yangpyeong-Goon, Republic of Korea; 4 Department of Rehabilitation Medicine, College of Medicine, Bucheon St. Mary’s Hospital, The Catholic University of Korea, Seoul, Republic of Korea; 5 Institute for Basic Medical Science, Catholic Medical Center, The Catholic University of Korea, Seoul, Republic of Korea

**Keywords:** Neuroscience, Neurology

## Abstract

Transcranial Direct Current Stimulation (tDCS) has benefits for motor rehabilitation in stroke patients, but its clinical application is limited due to inter-individual heterogeneous effects. Recently, optimized tDCS that considers individual brain structure has been proposed, but the utility thereof has not been studied in detail. We explored whether optimized tDCS provides unique electrode positions for each patient and creates a higher target electric field than the conventional approach. A comparative within-subject simulation study was conducted using data collected for a randomized controlled study evaluating the effect of optimized tDCS on upper extremity function in stroke patients. Using Neurophet tES LAB 3.0 software, individual brain models were created based on magnetic resonance images and tDCS simulations were performed for each of the conventional and optimized configurations. A comparison of electrode positions between conventional tDCS and optimized tDCS was quantified by calculation of Euclidean distances. A total of 21 stroke patients were studied. Optimized tDCS produced a higher electric field in the hand motor region than conventional tDCS, with an average improvement of 20% and a maximum of 52%. The electrode montage for optimized tDCS was unique to each patient and exhibited various configurations that differed from electrode placement of conventional tDCS. Optimized tDCS afforded a higher electric field in the target of a stroke patient compared to conventional tDCS, which was made possible by appropriately positioning the electrodes. Our findings may encourage further trials on optimized tDCS for motor rehabilitation after stroke.

## Introduction

Transcranial direct current stimulation (tDCS) noninvasively modulates cortical excitability through electrodes on the scalp that exchange weak direct currents. tDCS has shown beneficial effects on post-stroke motor rehabilitation, presumably by promoting cortical plasticity or restoring the interhemispheric balance^1,2^. tDCS has been widely used to treat several neurological disorders. However, high inter-subject variability in terms of the tDCS effects has hindered clinical applications in stroke patients^3,4^.

One major factor underlying the inconsistencies of tDCS effects is inter-individual anatomical variation^5^. Recent human studies and current flow simulations found that the distribution and intensity of the electric field induced by tDCS depended on individual brain anatomy and the conductivity of each tissue^6,7^. Anatomical variations, including scalp, skull, and cerebrospinal thicknesses, and cerebral cortex patterns, affect the amount of current attaining a target region, and thus are associated with suboptimal stimulation^6^. Brain structural changes in stroke patients, such as cerebrospinal fluid (CSF)-filled cavities or enlarged ventricles, also contribute to the heterogeneity of tDCS results. Computational modeling of stroke patients showed that the electric field distribution was altered by brain lesions; the overall electric field intensity in the cerebral cortex was significantly less than that in a healthy brain^8–10^. Therefore, personalized tDCS that considers individual anatomical structures and brain lesions is attracting increasing interest.

Despite the vast amount of research on tDCS, the relationship between stimulation intensity and response remains unclear because 'applied stimulation strength' and 'targeted stimulation strength' differ^11^. Advances in computational modeling have rendered it possible to predict the brain current flow for any given electrode. Simulation studies have reported that the electric field magnitude in the target area is associated with tDCS outcomes, suggesting that control of target stimulation is critical for optimal tDCS^12,13^. Stimulation can be increased by changing the current amplitude or duration^14^. However, the potential risks of brain and skin damage limit applications of high-dose currents. Bai et al. suggested that changing the electrode positions could “steer” the effect of tDCS by altering current flow in the brain^15^. Computational modeling has been used to optimize tDCS for stroke patients by adjusting the electrode positions, to maximize the electric field intensities in target areas^9,16,17^. One small pilot study showed that optimized tDCS in stroke patients with aphasia increased the electric field in the target area by 63% compared to that of conventional approaches^17^. Optimized tDCS is expected to be a promising treatment for recovery after stroke. However, there is a scarcity of additional studies investigating the distinctions in montage and electric fields between optimized tDCS designed for motor recovery in stroke patients and conventional tDCS. Such investigations can help prepare and conduct clinical trials to verify whether optimized tDCS is more effective at both behavioral and neurophysiological levels.

In this study, we hypothesized that optimized tDCS would feature a unique electrode positioning for each stroke patient and would create stronger electric fields at target sites than does conventional tDCS. We identified an optimized tDCS montage designed to maximize the electric field in hand motor areas, which are commonly targeted to improve upper limb function in stroke patients^18,19^. Using simulations, we compared the target electric field intensities of optimized and conventional tDCS, and defined factors associated with electrode positioning of optimized tDCS.

## Materials and methods

### Participants

This study was conducted in 21 hemiplegic stroke patients enrolled in the double-blinded, randomized controlled study to evaluate the effect of optimized tDCS on upper extremity function. Brief details regarding the clinical trial in which the patients analyzed in this study participated are as follows: Participants were recruited from St. Vincent’s Hospital and the National Traffic Injury Rehabilitation Hospital of the Republic of Korea from August 2021 to May 2023. Eligible patients were those aged 18 years or older with unilateral upper limb motor paralysis and at least 4 weeks after stroke onset. Participants were randomly assigned to three groups—optimized tDCS, conventional tDCS, and sham-tDCS. Active stimulation involved the application of a 2 mA current for 30 min in each session, administered once daily for a total of 10 sessions over 2 weeks. Assessments, including the Fugl-Meyer Assessment (FMA) to assess upper extremity function, were conducted at baseline and two weeks after the intervention. The full protocol has been described by Yoo et al.^20^. The study was reviewed and approved by the Institutional Review Boards of the Catholic University College of Medicine and the National Traffic Injury Rehabilitation Hospital. Written informed consent was obtained from all patients.

### Computational modeling

Neurophet tES LAB ver. 3.0 software was used to build individual head models using T1-weighted magnetic resonance imaging (MRI) scans of all patients and to simulate tDCS-induced electric fields^21^. Briefly, each head was segmented into cerebral and cerebellar gray and white matter, cerebrospinal fluid (CSF), the skull, the skin, and the stroke lesion^7^. A volume mesh head model was created based on the segmentation surface, and visualized. The generated meshes consisted of an average of 4,502,652 (SD = 344,194) tetrahedral elements (Supplemental Table1). The conductivities were: Gray matter 0.265 S/m; white matter 0.126 S/m; CSF 1.65 S/m; skull 0.010 S/m; skin 0.465 S/m; and stroke lesions 0.8087 S/m. Stroke lesions were distinguished from CSF by the different conductivities^22^.

**Table 1:** The descriptive characteristics of patients.

Age, median [IQR], years	59 [53–66]
Number of males (%)	14 (67)
Time from stroke onset to MRI, median [IQR], days	216 [49–460]
Number of subjects by stroke type	
Ischemic stroke	13
Hemorrhagic stroke	8
Number of subjects by lesion location	
Cortical	8
Subcortical	11
Brain stem	2
Initial FMA-UE, median [IQR]	21 [12.75–28.5]

*IQR* Interquartile range; *FMA-UE* Fugl-Meyer Assessment of the upper extremity.

Cortical—brain lesion involving cortical structures; Subcortical—a brain lesion involving only subcortical structures; Brain stem—a brain lesion involving only the brain stem.

### tDCS simulation

For conventional tDCS, electrode placement was determined using the 10–20 EEG system. The anode was placed over the M1 hand area (C3 or C4) of the affected hemisphere and the cathode over the contralesional M1 hand area (C4 or C3) for bi-hemispheric tDCS. The electrodes were modeled as 5 × 5-cm rectangular electrodes and 2 mA was delivered to the anode^23^. We formulated the tDCS problem by applying Maxwell’s equation, which was transformed into the Laplace equation to represent the tDCS conditions. The Laplace equation under static conditions is expressed as ∇∙∇V = 0 in Ω (Formula 1), where V represents the tDCS-induced potential, and Ω is the domain defined by the anatomical head model. The equation inherently meets the insulation condition at most outer boundaries, where this condition is fulfilled when the inner product between the normal vector and current density equals zero. Formula 1 was further defined using the finite element method (FEM). The Eigen library^24^ was employed to compute the tDCS-induced electric field with the conjugate gradient method.

For optimized tDCS, the coordinates of the target region were obtained by identifying the anatomical hand knob^25^ of the affected side in each brain MRI scan and entered into tES LAB software. The electrode type and the total current were those of conventional tDCS. We employed an algorithm specifically designed for a virtual grid to optimize the electrode location. Initially, the positions of candidate electrodes for optimization are defined in proximity to the reference electrode's position. These candidates form an evenly spaced grid, with 11.11 mm intervals, confined to the skin surface. Our algorithm performs a grid search and iterations to identify the optimal set of electrode candidates, maximizing the electric field strength within the specified region of interest (ROI).

### Electric field comparisons

The spatial distribution of electric fields derived from selected electrode montages was visualized using tES LAB software. The target ROI was defined as a three-dimensional (3D) sphere with a radius of 2 mm. The center coordinates (x, y, z) of the 3D sphere were determined by specifying a point within the hand knob area on each patient's brain MRI. The electric field strength presented in this study is the average value formed in the gray matter element within the specified ROI. The normal component of electric field at the target site was extracted to compare the results of the two tDCS simulations. The improvement on optimization was calculated as ((electric field during optimized tDCS minus electric field during conventional tDCS)/electric field during conventional tDCS) × 100%.

### Electrode montages

The electrode positions were quantitatively compared using the inter-electrode distances D, thus the Euclidean distances between conventional and optimized tDCS electrodes (Supplemental Fig. 1). D_anode_ (D_cathode_) are the distances from the centers of conventional tDCS anodes (cathodes) to the centers of optimized tDCS anodes (cathodes)*.* The sum of D_anode_ and D_cathode_ is the distance D. An increase in D means that the electrodes of the optimized tDCS are farther away from those of conventional tDCS. Additionally, the distance between the anode and cathode within each tDCS montage was calculated and compared.

**Figure 1:**
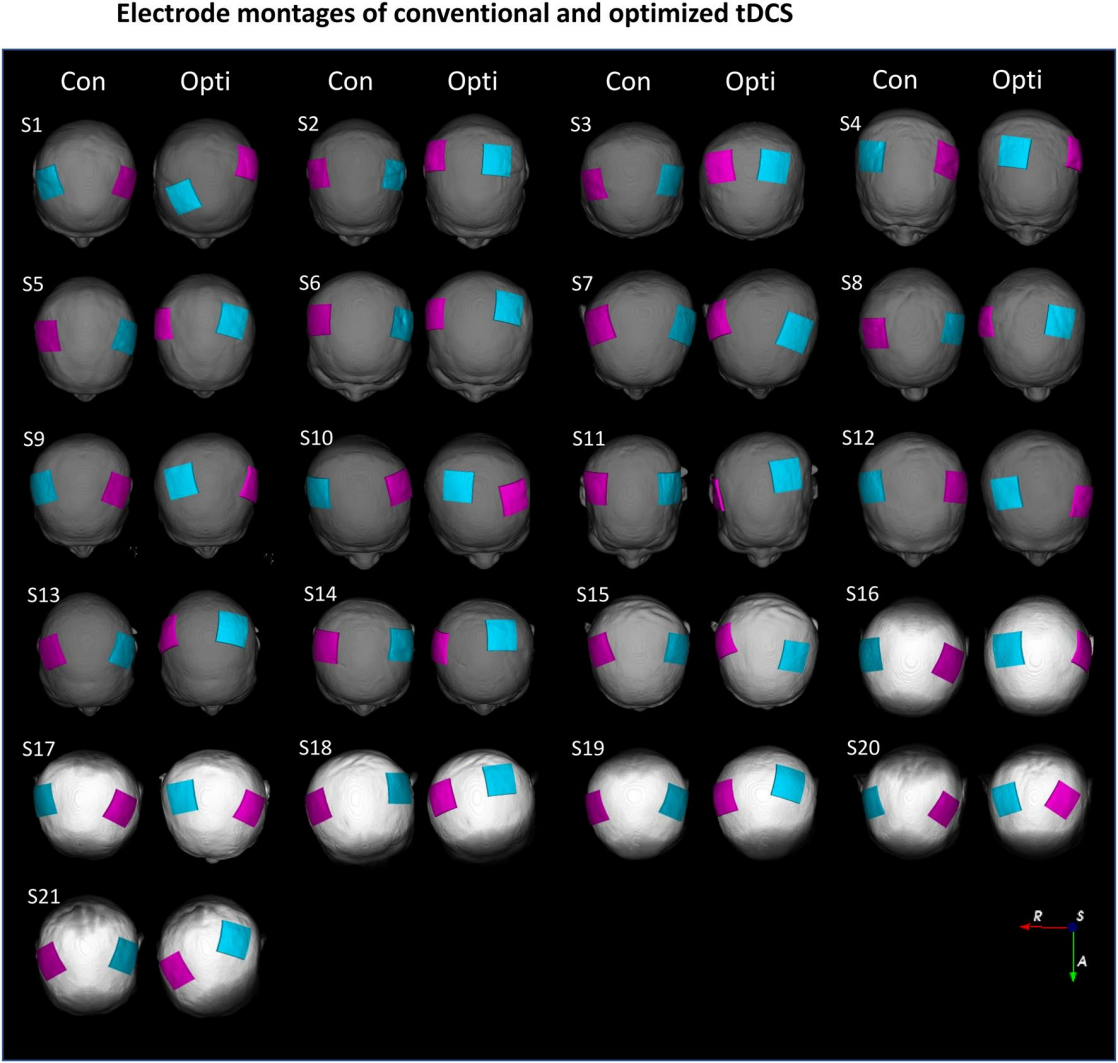
The electrode montages of conventional and optimized tDCSs. Montage pairs of conventional (left) and optimized (right) tDCSs determined via simulation for each patient displayed on individual head models. The pink electrode is the anode and the cyan electrode the cathode. Con—Conventional tDCS simulation; Opti—Optimized tDCS simulation; R—Right; A—Anterior; S—Superior.

### Statistical analysis

The Kolmogorov–Smirnov test was used to assess the normality of continuous variables. Non-normally distributed variables are described as medians with interquartile ranges [IQRs]. The Wilcoxon signed-rank test was used to compare the electric fields in target regions of conventional and optimized tDCS. The Mann–Whitney test was employed to investigate whether differences in the electric field or electrode position between conventional and optimized tDCS depended on brain lesion location. The relationships between D and the electric field, and the initial FMA-UE score, were analyzed by drawing scatterplots and calculating Spearman correlation coefficients. All statistical analysis employed MATLAB release 2021a (MathWorks Inc).

### Ethical approval

The study protocol was approved and reviewed by Institutional Review Board of Catholic University, College of Medicine (approval number. VC21DIDS0085), Institutional Review Board of National Traffic Injury Rehabilitation Hospital (approval number. NTRH-21004); This study was approved by the Ethics Committee of the Catholic University of Korea. The study was performed in accordance with the tenets of the Declaration of Helsinki, and all subjects provided written informed consent.

## Results

Descriptive characteristics are presented in Table 1. The median [IQR] age was 59 [53–66 ] years. The median [IQR] time from stroke onset to MRI was 216 [49–460 ] days. All stroke patients with cortical lesions exhibited infarctions in the middle cerebral artery territory.

### The electrode montages of conventional and optimized tDCS

The electrode configurations of conventional and optimized tDCS are shown in Fig. 1. During conventional tDCS, the anode and cathode were symmetrically placed at positions C3 and C4 of the 10–20 system. In the optimized tDCS, the electrode position varied greatly from patient to patient, although the hand knob was always the target. The electrode configurations for optimized tDCS differed even between patients with similar brain lesions. For example, patients S3 and S13 had similar infarctions in the right MCA territory, as revealed by MRI, but the electrode positions for optimized tDCS differed (Supplemental Figure 2). The median [IQR] of D between the conventional and optimized tDCS electrodes was 60.21 [57.14–66.94] mm; the anode-to-anode distance (D_anode_) 23.28 [17.60–29.99] mm and the cathode-to-cathode distance (D_cathode_) 37.87 [31.19–41.76] mm (Supplemental Table 2). D_cathode_ was significantly greater than D_anode_ (*P* < 0.001). The highest D value was 80.81 mm and the smallest 36.84 mm. The anode-to-cathode distance within the optimized tDCS montage was significantly shorter than that of the conventional montage (*p* < 0.001). The median [IQR] anode-to-cathode distance was 128.88 [124.33–132.72] mm for the conventional montage and 114.37 [107.24–122.84] mm for the optimized montage.

**Figure 2:**
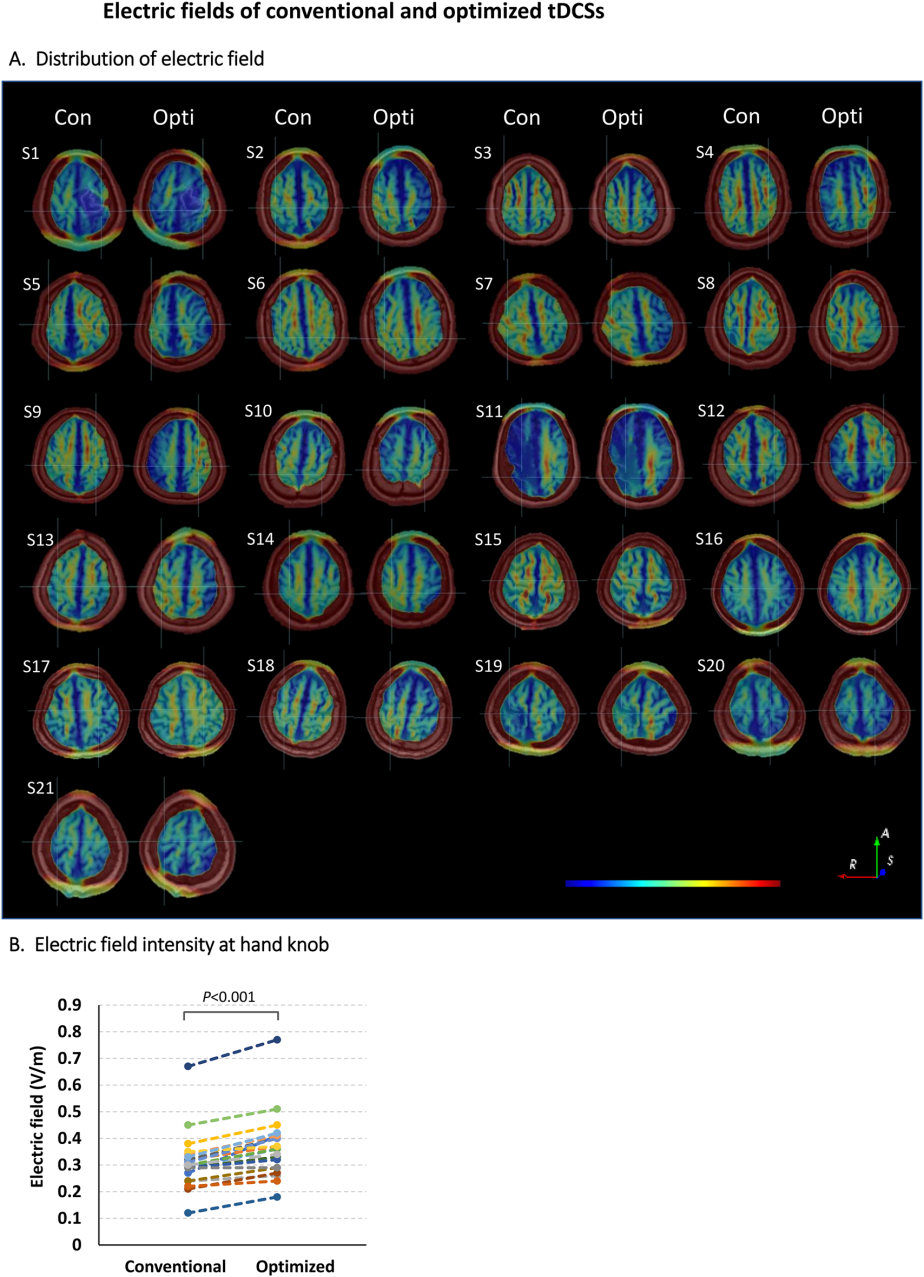
The electric fields of conventional and optimized tDCSs. (**A**) Electric field distributions at the target areas (the hand knobs) of conventional tDCS (left) and optimized tDCS (right) in 21 stroke patients. The centers of the intersecting lines correspond to the target areas. The magnitude of the electric field increases linearly from blue to red. Notation: Con, Conventional tDCS simulation; Opti, Optimized tDCS simulation; R—Right; A—Anterior; S—Superior. (**B**) The electric field intensities in the target regions of conventional and optimized tDCS simulations. Circles represent individual patient data.

### The electric field of conventional and optimized tDCS

Axial sectional images of the electric field distributions of the two tDCS simulations for each patient's head model are shown in Fig. 2A. During conventional tDCS, the electric field is symmetrically distributed over both hemispheres, mainly centered on the superior frontal gyrus. On the other hand, in the optimized tDCS, the distribution of the electric field is skewed around the target area, the hand knob. The magnitudes of the electric fields in the target regions are summarized in Supplemental Table 3. The conventional montage generated a median [IQR] electric field intensity of 0.30 [0.26–0.33] V/m at the hand motor cortex. The optimized tDCS field was 0.36 [0.29–0.41] V/m, significantly larger than the conventional tDCS field in all but one patient (*P* < 0.001) (Fig. 2B). Compared to the conventional montage, the optimized montage demonstrated an average 20% improvement in electric field strength, with the maximum enhancement reaching up to 52%.

### Factors associated with electrode positioning of the optimized tDCS

**Figure 3:**
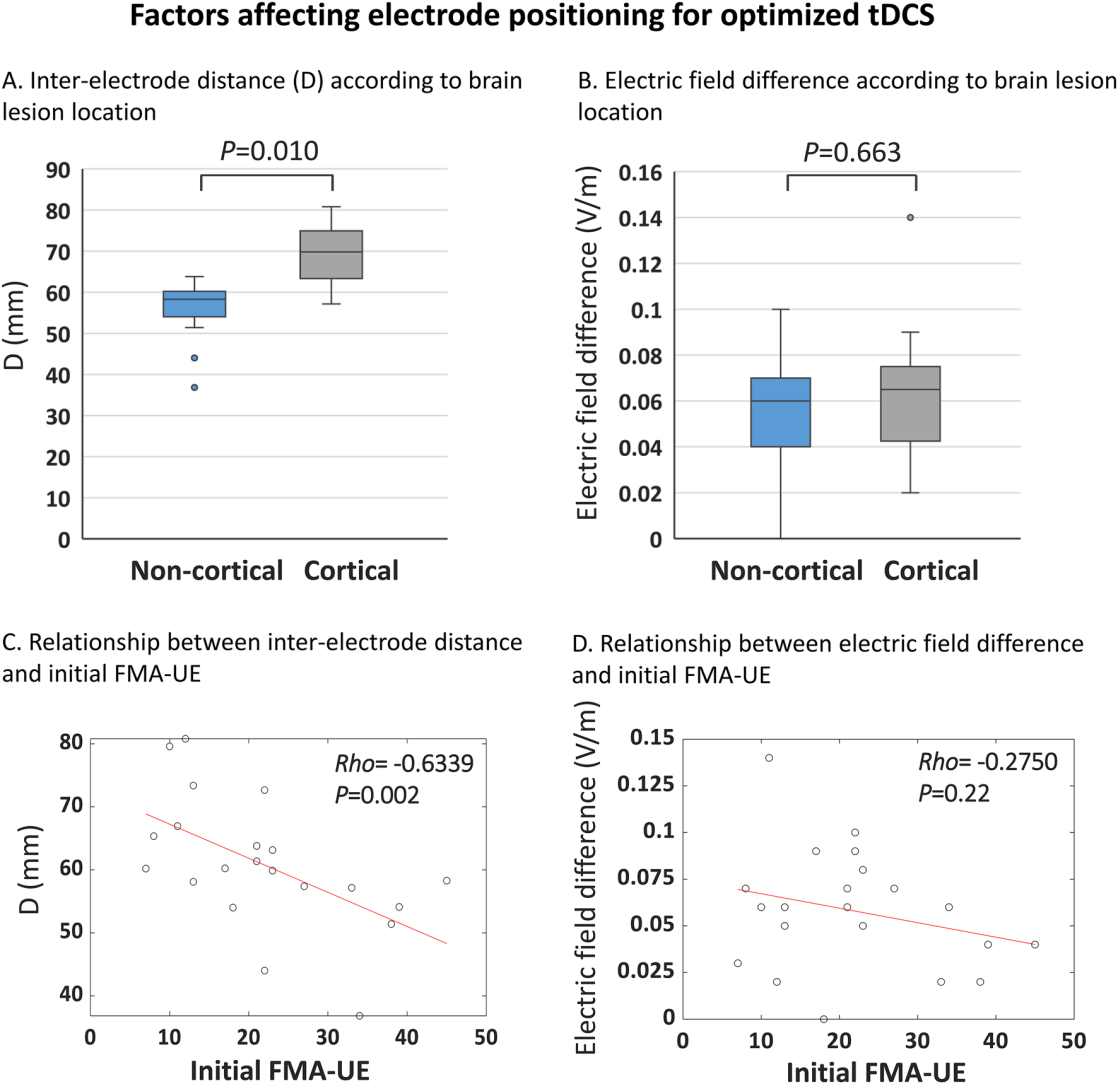
Factors affecting electrode positioning for optimized tDCS. (**A**) The distances (Ds) between conventional and optimized tDCS electrodes in groups with cortical and non-cortical lesions. (**B**) The target electric field differences between conventional and optimized tDCSs. The midlines of the boxes indicate the medians of A and B, the ends of the boxes the interquartile ranges (25–75%), and the whiskers the minima and maxima. (**C**) Correlations between the Ds and the initial Fugl-Meyer Assessment of upper extremity (FMA-UE) scores. The best-fitting regression lines are superimposed. (**D**) Correlations between the target electric field differences of the two tDCSs and the initial FMA-UE scores. *Rho*: Spearman correlation coefficient.

In some patients, the electrode positions of the optimized tDCS differed greatly from those of the conventional tDCS, and *D*, the sum of the distances between the tDCS electrodes, increased. To determine whether electrode positioning of the optimized tDCS was affected by stroke lesion location, we compared patients with cortical and non-cortical brain lesions. D was larger in the former group (median [IQR] non-cortical 58.28 [53.35–60.51] mm; cortical 69.80 [61.37–76.49] mm, *P* = 0.010) (Fig. 3A). The IQR was also greater in the group with cortical lesions. The difference in target electric field intensity between conventional and optimized tDCS were not affected by brain lesion location (Fig. 3B). The scatterplot revealed a strong negative correlation between D and the initial FMA-UE score (Spearman *Rho* –0.63; 95% confidence interval –0.83 to –0.34; *P* = 0.002; Fig. 3C). The difference in electric field magnitude between the two tDCS were not associated with initial FMA-UE score (Fig. 3D). There was also no correlation between target electric field intensity and initial FMA-UE score in both optimized and conventional tDCS (Supplemental Figure 3). The shortening of the anode-to-cathode distance of the optimized montage, compared to the conventional one, showed no correlation with the location of brain lesions or initial FMA scores. The electrode positions of the optimized tDCS were not affected by age, sex, or the time since stroke (data not shown).

## Discussion

We hypothesized that computational modeling might place electrodes in positions different to those of conventional tDCS, thus increasing the electric field in the target region. We simulated conventional and optimized tDCSs for 21 stroke patients using a within-subject design, and compared the results. Optimized tDCS generated higher-intensity electric fields at targets than did conventional tDCS. Electrode placement for optimized tDCS varied greatly among patients, and differed from the conventional placement. In patients with cortical lesions or low initial FMA-UE scores, the electrode positions of optimized tDCS were remote from those of conventional tDCS. The results confirm our hypothesis; tDCS optimized via computational modeling may improve stroke motor rehabilitation to a greater extent than does conventional tDCS.

Computational modeling using a healthy head model showed that conventional tDCS delivered a robust, average electric field near the hand motor area^12^. However, application of a conventional montage to the stroke head model significantly reduced the electric field in the hand motor region and triggered unexpected current flow caused by the lesion^8,17^. To overcome the limitations of conventional tDCS, we designed an optimized montage that produced the maximum electric field in the hand knob. The positions of the optimized tDCS electrodes differed from those of conventional electrodes, and the inter-subject variance was large. This result is in agreement with a recent study showing that the optimal anode position targeting hand knob is primarily C3 in healthy individuals, but a different position in stroke patients^9^. However, in the study by van der Crujan et al., the optimal electrode positions were not various because the candidate electrodes were limited to the 80 electrodes of a 10/10 system. On the other hand, in our current study, the optimal electrode position was determined using a more sophisticated 11.11 mm spacing grid, revealing the diversity of individual, optimal electrode positions. Our results suggest that standard, single electrode configurations do not ensure effective stimulation of stroke patients, and that personalized montages that consider the brain lesions and anatomical differences can be a useful strategy for stroke rehabilitation via tDCS.

We found that the differences in electrode positions between conventional and optimized tDCS were greater in those with cortical than non-cortical lesions, suggesting that the former lesions significantly affect electric field and increase complexity when computationally modeling optimal montage selection. In patients with low initial FMA-UE scores, the optimized electrodes lay further away from conventional electrodes. Strokes that damage the corticospinal tract are accompanied by reductions in the thickness and surface area of the associated cerebral cortex^26,27^. Cortical atrophy and the resulting increase in local CSF thickness greatly reduce tDCS induced-electric field^8^. In patients with low FMA-UE scores, the corticospinal tract may be seriously damaged and the associated cortical structures changed, affecting the optimal tDCS electrode configuration by altering the electric field around the hand motor area. Therefore, when treating stroke patients with cortical lesions or severely compromised initial upper limb function, use of an optimal montage determined via computational modeling will enhance the effectiveness of tDCS.

Dmochoswki et al. developed an optimal tDCS montage using a volume conduction model and high-definition (HD) electrodes, increasing the electric field strength by an average of 63% compared to the conventional approach^17^. In our present study, the electric field magnitude increased by the optimized tDCS averaged about 20%. This lower increase may be due to our use of large electrodes. A large electrode generates wide but rather weak electric fields; HD electrodes create concentrated strong fields. While HD-tDCS can provide higher focality, we chose bi-hemispheric stimulation using traditional electrodes to optimize tDCS for stroke patients. In the context of stroke recovery, tDCS is commonly applied within the framework of the interhemispheric competition model. This model assumes that post-stroke, the overactive unaffected hemisphere exerts inhibitory influence over the hypoactive affected hemisphere^28^. Bi-hemispheric tDCS was chosen based on the premise that it can enhance affected cortical excitability through anodal stimulation while concurrently reducing unaffected cortical excitability through cathodal stimulation. Despite recent controversies surrounding the interhemispheric competition model^29^, numerous randomized controlled trials (RCT) have demonstrated the effectiveness of bi-hemispheric tDCS using traditional large electrodes in improving upper extremity function in stroke patients^30,31^. Furthermore, a recent meta-analysis comparing three stimulation types (anodal tDCS, cathodal tDCS, and bi-hemispheric tDCS) revealed that bi-hemispheric tDCS exhibited a relatively large effect size in promoting motor recovery of paralyzed upper extremities^18^. In contrast, there is currently no study verifying the effect of HD-tDCS on the recovery of upper extremity function in stroke patients through RCT. Given these findings, it is reasonable to employ validated bi-hemispheric tDCS to best achieve the goal of our clinical trial: assessing the effectiveness of electrode optimization in stroke rehabilitation.

The electric field strength required to produce a physiological effect is unknown, but one study found that a weak electric field of 0.2 V/m induced small but coherent changes in neuronal timing and rate changes, by affecting dynamic network activity^32^. During in vivo human intracranial recordings, a 1-mA tDCS produced a maximal electric field of 0.4 to 0.5 V/m in the cortical brain, close to the value predicted by computational modeling^6,7^. One meta-analysis found that a 1-mA tDCS significantly increased corticospinal excitability and that tDCS efficacy depended on current density^33^. These reports indicate that electric field increases of less than 0.1 V/m may affect the tDCS outcomes. Whether the optimized tDCS developed here really improves upper extremity function will be revealed by a randomized clinical trial that is currently in progress^20^.

In this study, the MRI segmentation involved only six tissue compartments. Some studies opt for a more detailed tissue segmentation to create realistic head models. Incorporating multiple tissues in modeling can enhance the accuracy of electric field estimation for tDCS, but it also introduces uncertainty to the simulation when the conductivity of the tissues is not precisely known^34^. Following a foundational guideline for head modeling^35^, we included the standard three tissues (brain, skull, and skin) along with the highly conductive CSF in the segmented tissues. Additionally, we made the distinction between gray and white matter, which has a significant impact on modeling results. Finally, our head modeling was aligned with the recently validated FEM model through in vivo recordings, thereby reinforcing the validity of our methodology^7^. In terms of head modeling, our study did not consider white matter anisotropy as a physical property. White matter tissues are highly anisotropic in their electrical conductivity, which can exert a substantial influence on electric field directionality and spatial distribution during tDCS^36^. However, the effect on cortical electric fields is not significant^37,38^. A recent study indicated that incorporating white matter anisotropy did not enhance model accuracy^7^. The inclusion of white matter anisotropy requires the acquisition of diffusion tensor imaging data, a process that is time-consuming and costly^11^. Therefore, given the current evidence, incorporating white matter anisotropy into tDCS electric field simulations may not be a cost-effective strategy. Future studies exploring the effects of white matter anisotropy in stroke brain models will be needed.

Our study had several limitations. Optimized tDCS generated higher electric fields in target regions than did conventional tDCS in most patients. However, for patient S20, there was no difference. The subject in question was a chronic patient with basal ganglia hemorrhage and showed no notable findings. Out of the 21 patients, four exhibited less than a 10% improvement in the electric field by optimized tDCS. This poor response was not related to the location or type of brain lesion, initial FMA score, chronicity, or age. It is postulated that the extent of improvement in the electric field is linked to anatomical variations in the current path during tDCS, yet the specific contributing factor remains unidentified. Another issue in our study is the uncertainty of stroke lesion conductivity. Several studies used the CSF value, but this may be inaccurate^8,9^. In this study, we manually segmented stroke lesions distinct from liquefaction lesions on MRI. The conductivity of the lesion was specified as 0.8087, which is the average of reference values for several known brain lesions^22^. Regarding the uncertainty of stroke lesion conductivity, future research should explore sensitivity studies to investigate the impact of changes in stroke lesion conductivity on the electric field. An additional limitation in our study is the presentation of the electric field distribution for two tDCS setups solely through cross-sectional graphic images, without providing specific numerical values associated with the electric field distribution. Many studies have demonstrated the significance of the magnitude among electric field characteristics, revealing its substantial association with the neurophysiological, behavioral, or functional outcomes of tDCS^39–41^. Consequently, our study delved into the analysis of tDCS simulation results with a primary focus on the strength of the electric field. However, several studies have indicated that estimates of electric field distribution, as predictors of tDCS response, can elucidate the variability in individual responses to tDCS^42^. The quantitative analysis using variables such as field focality values can enhance our understanding of the characteristics of optimized tDCS and contribute to the interpretation of potential differences in clinical outcomes between the two tDCS approaches. As part of our future plans, we intend to conduct further analysis of electric field distribution after the completion of our clinical trial.

## Conclusion

We demonstrated that the electric field induced by tDCS was improved after optimization. Optimized tDCS may benefit stroke patients with structural brain changes that impede the production of adequate electric fields. Our findings will contribute to the development of optimized tDCS protocols for clinical applications.

### Supplementary Information

Supplementary Information.

## Data Availability

The datasets generated and/or analysed during the current study are not publicly available due on-going clinical trial but are available from the corresponding author on reasonable request.
