# Supplementary material for: Electric field simulation and appropriate electrode positioning for optimized transcranial direct current stimulation of stroke patients: an in Silico model
Source: Sci Rep. 2024 Feb 3;14:2850. doi: 10.1038/s41598-024-52874-y (PMC10838316; doi:10.1038/s41598-024-52874-y)
Supplement: Supplementary file 1 — Supplementary Information. [file 41598_2024_52874_MOESM1_ESM.docx]

**Supplemental Figure 1. Calculation of the inter-electrode distance, D**

**Supplemental Figure 2. Optimized electrode montage for subject S3 and S13**

**Supplemental Figure 3. No correlation of target electric field induced by tDCS and initial FMA-UE score**

**Supplemental Table 1. Number of mesh elements for each head model**

**Supplemental Table 2. Distance between conventional tDCS electrodes and optimized tDCS electrodes**

**Supplemental Table 3. Electric field intensity at target region in conventional and optimized tDCS**

**Supplemental Figure 1. Calculation of the inter-electrode distance, D**

**
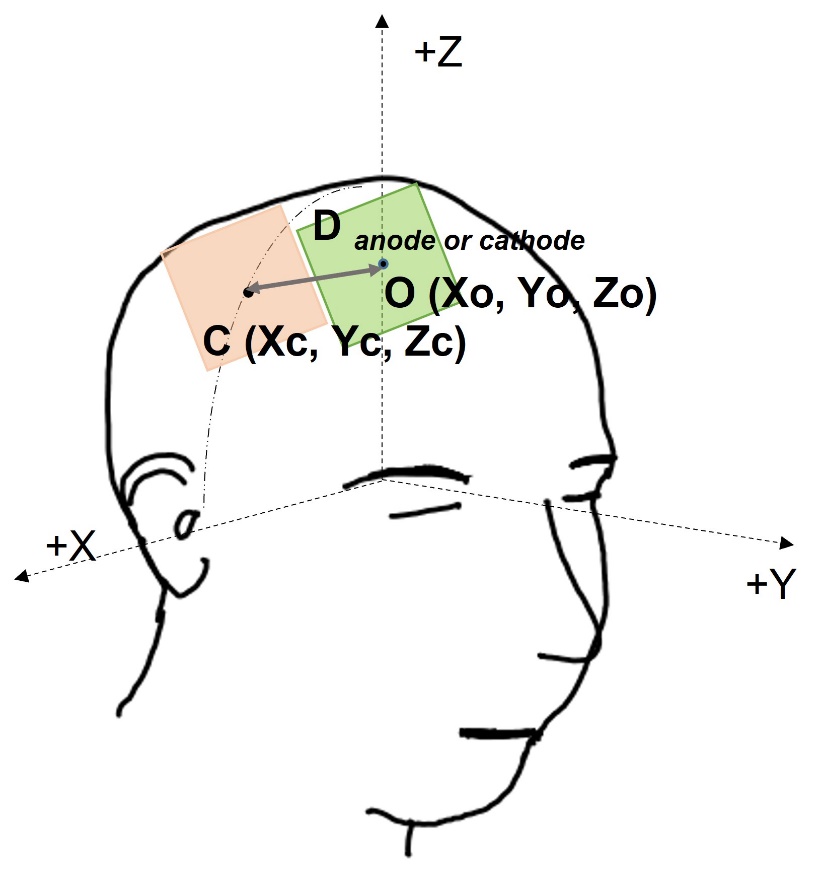
**

The orange electrode is the conventional tDCS electrode, and the green electrode is the optimized tDCS electrode. The inter-electrode distance, *D* was calculated using the following formula

D= $\sqrt{({X{c-Xo)}^{2}+{(Yc-Yo)}^{2}+(Zc-Zo)}^{2}}$,

where Xo, Yo, and Zo are NIfTI coordinate values for the center of the optimized tDCS electrode, and Xc, Yc, and Zc are NIfTI coordinate values for the center of the conventional tDCS electrode. D*_anode_* represents the Euclidian distance from the center of the conventional tDCS anode to the center of the optimized tDCS anode*.* D*_cathode_* represents the Euclidian distance from the center of the conventional tDCS cathode to the center of the optimized tDCS cathode. The sum of D*_anode_* and D*_cathode_* is defined as the distance D, which represents the discrepancy of electrode positions between conventional tDCS and optimized tDCS.

**Supplemental Figure 2. Optimized electrode montage for subject S3 and S13**


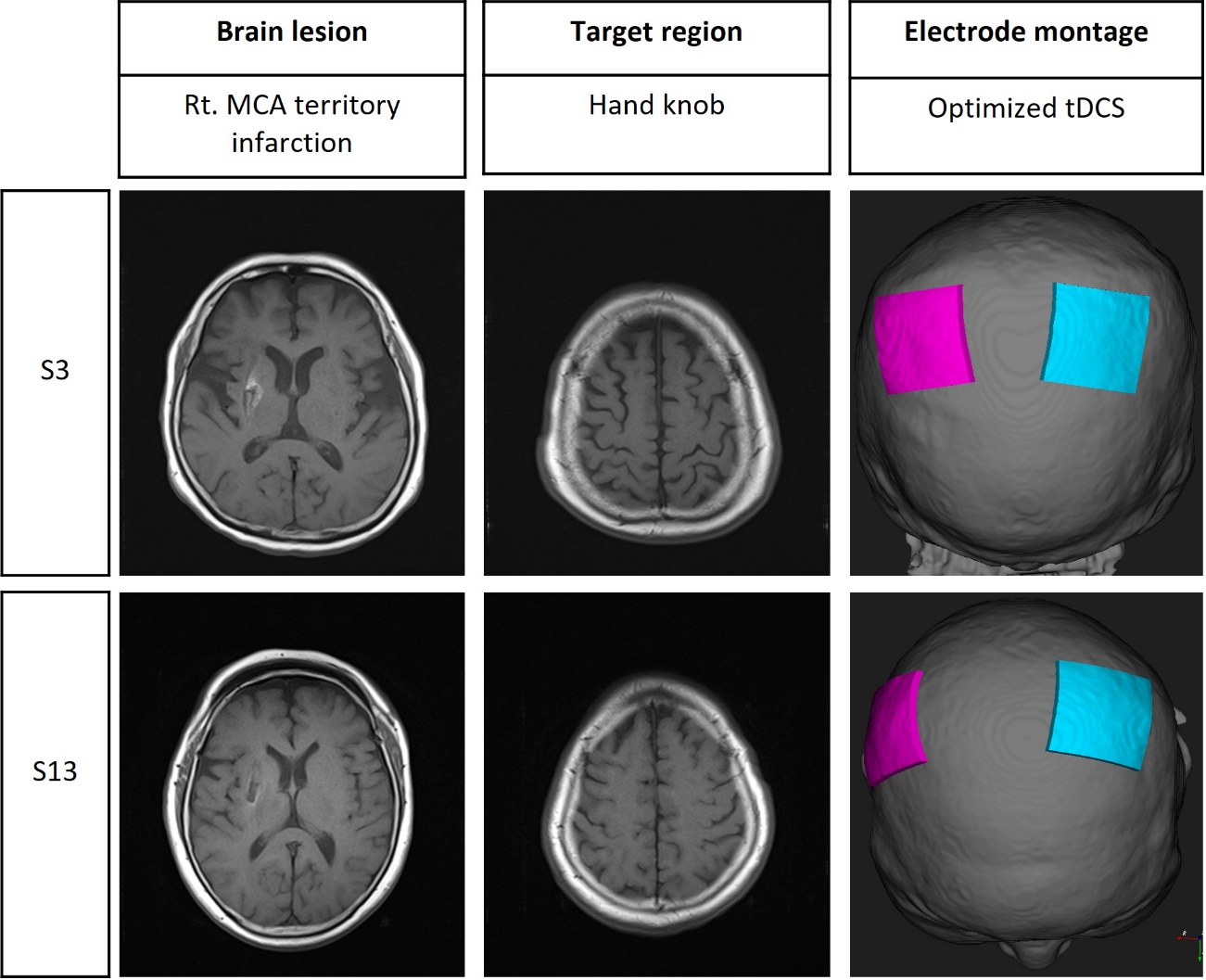


Left column shows MRI image of scar remaining after right middle cerebral artery territory infarction. Middle column shows MRI image corresponding to the hand knob area, which is the target of tDCS. Right column shows the montage pairs of optimized tDCS. The pink electrode represents the anode and the cyan electrode represents the cathode.

**Supplemental Figure 3. No correlation of target electric field induced by tDCS and initial FMA-UE score**


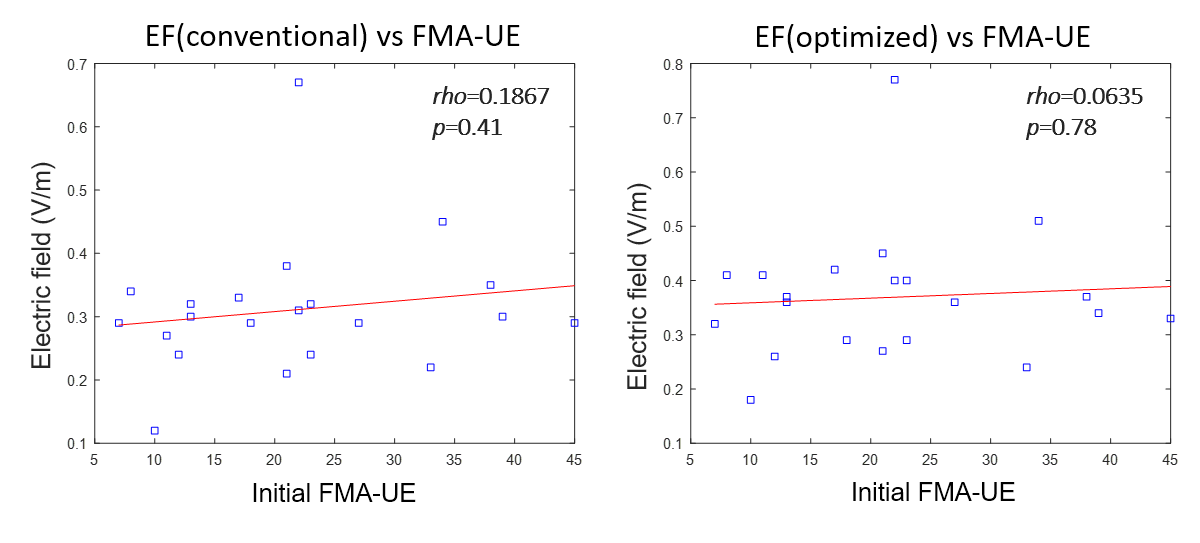


**Supplemental Table 1. Number of mesh elements for each head model**

| **Patient** | **Mesh elements** |
| --- | --- |
| S1 | 4,733,814 |
| S2 | 4,950,951 |
| S3 | 4,657,418 |
| S4 | 4,721,610 |
| S5 | 4,388,246 |
| S6 | 4,751,685 |
| S7 | 4,250,296 |
| S8 | 4,669,387 |
| S9 | 3,912,159 |
| S10 | 4,772,203 |
| S11 | 4,163,605 |
| S12 | 4,663,435 |
| S13 | 3,750,594 |
| S14 | 4,158,070 |
| S15 | 4,752,094 |
| S16 | 4,324,572 |
| S17 | 4,626,978 |
| S18 | 5,001,684 |
| S19 | 4,387,535 |
| S20 | 4,816,512 |
| S21 | 4,102,843 |
| **Average**  **[SD]** | 4,502,652  [344,194] |

**Supplemental Table 2. Distance between conventional tDCS electrodes and optimized tDCS electrodes**

| **Patient** | **D*_cathode_*** (mm) | **D*_anode_*** (mm) | **D** (mm) |
| --- | --- | --- | --- |
| S1 | 34.76 | 32.19 | 66.95 |
| S2 | 46.40 | 26.98 | 73.38 |
| S3 | 44.44 | 36.37 | 80.81 |
| S4 | 38.24 | 23.13 | 61.37 |
| S5 | 37.24 | 20.16 | 57.39 |
| S6 | 41.45 | 16.66 | 58.10 |
| S7 | 27.89 | 16.12 | 44.02 |
| S8 | 32.60 | 31.21 | 63.80 |
| S9 | 32.12 | 27.76 | 59.89 |
| S10 | 44.69 | 18.46 | 63.15 |
| S11 | 37.88 | 41.72 | 79.60 |
| S12 | 28.87 | 29.42 | 58.29 |
| S13 | 41.67 | 30.99 | 72.66 |
| S14 | 42.07 | 23.28 | 65.35 |
| S15 | 31.41 | 19.99 | 51.40 |
| S16 | 30.57 | 29.67 | 60.23 |
| S17 | 29.56 | 7.28 | 36.84 |
| S18 | 42.43 | 17.77 | 60.20 |
| S19 | 40.05 | 17.10 | 57.15 |
| S20 | 27.96 | 26.04 | 54.01 |
| S21 | 38.63 | 15.48 | 54.12 |
| **median**  **[IQR]** | 37.87  [31.19-41.76] | 23.28  [17.60-29.99] | 60.21  [57.14-66.94] |

D*_anode_* represents the Euclidian distance from the center of conventional tDCS anode to the center of optimized tDCS anode*.* D*_cathode_* represents the Euclidian distance from the center of conventional tDCS cathode to the center of optimized tDCS cathode. D is the sum of D*_cathode_* and D*_anode_*. IQR, interquartile range.

**Supplemental Table 3. Electric field intensity at target region in conventional and optimized tDCS**

| **Patient** | **Conventional EF (V/m)** | **Optimized EF (V/m)** | **Improvement (%)** |
| --- | --- | --- | --- |
| S1 | 0.27 | 0.41 | 52 |
| S2 | 0.32 | 0.37 | 16 |
| S3 | 0.24 | 0.26 | 8 |
| S4 | 0.38 | 0.45 | 18 |
| S5 | 0.29 | 0.36 | 24 |
| S6 | 0.3 | 0.36 | 20 |
| S7 | 0.67 | 0.77 | 15 |
| S8 | 0.21 | 0.27 | 29 |
| S9 | 0.32 | 0.40 | 25 |
| S10 | 0.24 | 0.29 | 21 |
| S11 | 0.12 | 0.18 | 50 |
| S12 | 0.29 | 0.33 | 14 |
| S13 | 0.31 | 0.40 | 29 |
| S14 | 0.34 | 0.41 | 21 |
| S15 | 0.35 | 0.37 | 6 |
| S16 | 0.33 | 0.42 | 27 |
| S17 | 0.45 | 0.51 | 13 |
| S18 | 0.29 | 0.32 | 10 |
| S19 | 0.22 | 0.24 | 9 |
| S20 | 0.29 | 0.29 | 0 |
| S21 | 0.30 | 0.34 | 13 |
| **median [IQR]** | 0.30  [0.26-0.33] | 0.36  [0.29-0.41] | *P* value <0.001 |

Improvement (%) was calculated as ((electric field during optimized tDCS minus electric field during conventional tDCS)/electric field during conventional tDCS) x 100%. *P* value was derived from Wilcoxon signed rank test. IQR, interquartile range, EF, electric field intensities.
